# Cation-π Bonding in Actinides: UO_*x*_^+^(Benzene) (*x* = 0, 1, 2) Complexes Studied with Threshold Photodissociation Spectroscopy and Theory

**DOI:** 10.1021/acs.jpclett.4c03603

**Published:** 2025-02-03

**Authors:** Jason E. Colley, Anna G. Batchelor, B. Wade Stratton, Michael A. Duncan

**Affiliations:** † Department of Chemistry, University of Georgia, Athens, Georgia 30602, United States

## Abstract

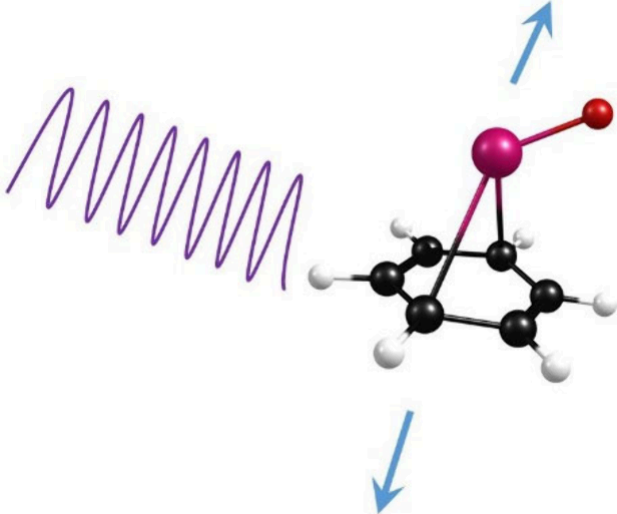

Cation-π complexes of the form UO_*x*_^+^(benzene) (*x* = 0, 1, 2) are produced by laser vaporization and cooled in a supersonic molecular beam. These ions are mass selected and studied with UV–visible laser photodissociation spectroscopy. Each of these complexes photodissociates by elimination of the benzene ligand. Above an energetic threshold, the absorption and photodissociation are continuous, indicating a high density of strongly coupled electronic states. The thresholds for the dissociation of each of these three complexes are measured and assigned as their respective bond dissociation energies. The bond energies determined [U^+^–(benzene): 42.5 ± 0.3 kcal/mol; UO^+^–(benzene): 41.0 ± 0.3 kcal/mol; UO_2_^+^–(benzene): 39.7 ± 0.3 kcal/mol] are comparable to those of transition metal ion-benzene complexes. Computational studies at the DFT/B3LYP level complement the experiments, predicting dissociation energies in reasonably good agreement with the experiments. Experiments and theory agree that the U^+^(benzene) complex is more strongly bound than its corresponding oxide ions. This new thermochemistry on actinide cation-π bonding should stimulate higher-level computational studies on these systems.

Cation-π bonding is a central ingredient of organometallic chemistry, biochemistry, and catalysis.^1−7^ Organometallic chemistry usually features the transition metals which form stable π complexes with many arene systems.^6,7^ Organometallics involving the actinide (An) metals are less common, although species such as An(Cp)_4_ (Cp = cyclopentadiene) and bis-cyclooctatetraene uranium (“uranocene”) are well-known.^8−13^ Gas phase ion chemistry has been employed for many years to investigate the reactions, thermochemistry and spectroscopy of transition metal organometallics in the absence of solvents or counterions,^14−39^ and these methods have been applied to actinide systems.^40−45^ In the present report, we use laser photodissociation spectroscopy of mass-selected ions to investigate the bonding energetics in U^+^(benzene), UO^+^(benzene) and UO_2_^+^(benzene) complexes, providing new thermochemistry for actinide cation-π bonding.

The most common approach used to investigate cation-molecular bond energies is collision-induced dissociation, and this method has been employed for many transition metal ion-benzene systems.^16,21,35^ Photodissociation methods have also been applied recently to these systems,^15,19,30,34,36−39,43^ including photofragment imaging^34,36−38^ and tunable laser measurements of photodissociation thresholds.^37,38^ Unfortunately, these approaches have not been applied extensively to actinide complexes. Actinides such as uranium have unpaired *5f* electrons and many low-lying electronic states which provide challenging problems for both experiments and theory. Benchmark physical properties such as bond energies are essential to evaluate the capabilities of theory and to refine its performance.

Computational chemistry of actinide systems has been pursued for many years, revealing the severe complexity of the electronic structure of these systems.^46−63^ Several different theoretical approaches have been pursued with which to handle the multielectron, multireference, relativistic and spin–orbit problems for actinide metal-molecular bonding. The performance of these computational methods have been tested with spectroscopy of atomic species, small actinide diatomics, and some larger cation-molecular complexes.^64−88^ One particularly interesting approach has been that of Dolg, Petersen and others using density functional theory (DFT) combined with an appropriate core potential and correlation-consistent basis set.^51,53,56^ The cc-pVTZ-PP basis set is designed to be used together with the Stuttgart/Köln 60 electron relativistic effective core potential (ECP60MDF). This approach has been shown to work reasonably well for several systems, including the vibrational spectra of uranium cation complexes.^84^ Here, we use this method to examine the bonding energetics of uranium and uranium-oxide ion complexes with benzene.

Figure 1 shows the mass spectrum produced upon laser vaporization of a depleted uranium rod in an expansion of helium seeded with benzene vapor at ambient temperature. As indicated, ions of the form UO_*x*_^+^(benzene)_*y*_ (x = 0,1,2; y = 1,2) are formed. Although no oxygen is added to the expansion gas, the oxide ions in this system are always present because of partial oxidation of the uranium rod surface. When these ions are mass-selected and excited with visible laser wavelengths, photodissociation occurs efficiently, eliminating a neutral benzene ligand. At higher energies in the UV (e.g., 355 nm), photodissociation of U^+^(benzene) produces U^+^(C_4_H_4_) and U^+^(C_2_H_2_) ions in addition to the U^+^ fragment, as shown in previous work.^43^ In that same study, UV photodissociation of the monoxide- and dioxide-benzene cations was found to eliminate only the intact benzene ligand.^43^

**Figure 1:**
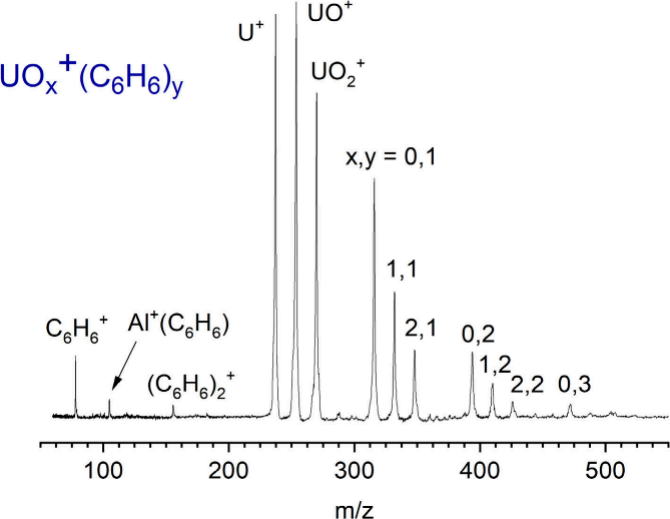
Mass spectrum of uranium-benzene and uranium oxide-benzene ions produced by laser vaporization.

Wavelength scans of the photodissociation show that a minimum energy is required for photodissociation, but above this threshold the spectrum is unstructured and continuous. This is not surprising because the manifold of excited electronic states for U^+^, UO^+^ and UO_2_^+^ is extremely dense,^65−72^ leading to an even greater density of states for the corresponding cation-benzene complexes. This state density and its role in predissociation spectroscopy measurements has been discussed previously in the case of neutral uranium molecules.^88^ In such a situation, when the density of excited states is great enough to be essentially continuous, absorption occurs at almost all wavelengths, spin–orbit interactions and nonadiabatic interactions couple these states to each other, and photodissociation can occur as soon as the photon energy exceeds the bond dissociation energy. This kind of threshold photodissociation (TPD) measurement and its assignment to the bond energy was described first by Smalley and co-workers for transition metal ions.^89^ Later, it was employed by several other groups.^37,38,90−96^ We applied this approach in recent studies of Fe^+^(acetylene) and Fe^+^(benzene) complexes.^37,38^ This method is closely related to that using the predissociation threshold in REMPI signals described by Morse and co-workers.^88,97−100^

Figure 2 shows the wavelength scan of the threshold for photodissociation of the U^+^(benzene) complex. The ions were produced in an argon expansion to enhance cooling. The parent ion was mass selected in a reflectron time-of-flight spectrometer and excited with the tunable output from a UV–visible OPO laser system. The intensity of the U^+^ ion resulting from elimination of benzene was recorded as a function of the laser wavelength. The laser pulse energy was adjusted to a level of about 1.0 mJ/pulse to avoid multiphoton absorption. The noise level in the experiment is mainly from the shot-to-shot intensity fluctuations in the parent ion signal. We find that laser ablation of uranium is efficient, but noisy because of the irregular partial oxidation of the metal rod surface. We use an approximate linear fit of the baseline and of the rising threshold to account for this noise, and assign the threshold at the intersection of these lines. This results in the assignment of the threshold at 672 ± 5 nm. The threshold values and the error bars here are estimated from the noise levels in the spectra and the variation of the onset in the multiple different threshold scans conducted for each of these ions. This wavelength corresponds to a photon energy of 1.85 ± 0.02 eV or 42.6 ± 0.3 kcal/mol. This energy is therefore a strict upper limit on the U^+^–benzene bond energy. If the density of electronic states and absorption is continuous, which appears to be true, then this threshold corresponds to the actual bond energy.

**Figure 2:**
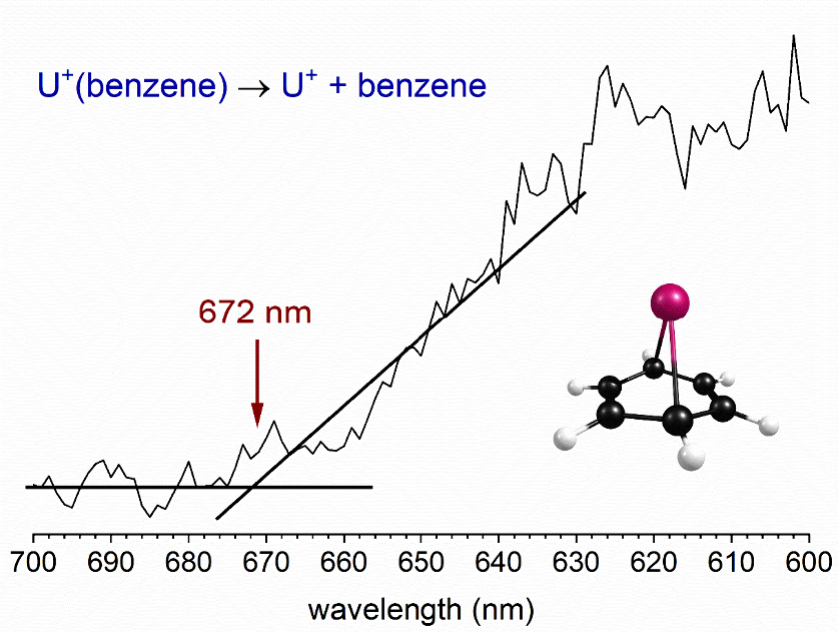
Threshold photodissociation spectrum of U^+^(benzene) measured in the U^+^ fragment ion channel.

A threshold such as this can vary with the temperature of the ions. If the ions have internal energy, then the threshold could appear at a lower energy than the true bond energy. We in fact found this to be true when these ions were produced in a helium expansion. To investigate cooling for systems such as this, we have previously employed different expansion gases that might have better or worse collisional cooling. For several previous systems, expansions in argon, N_2_ or CO_2_ produced the same results. We therefore we conclude that argon expansions achieve the best cooling possible and that is why we use it for the present experiments. The exact temperature is unfortunately not possible to measure. But in other similar systems where rotational structure could be measured, temperatures were in the 10–50K range.^101,102^ Although vibrational temperatures can be higher than rotational temperatures, we believe that this range is also likely for the ions in this experiment. In the case of the Fe^+^(acetylene) and Fe^+^(benzene) complexes studied recently by our group, the bond energies derived from the scanned photodissociation thresholds for ions expanded in argon matched the previous results from collision-induced dissociation experiments, validating this method.^37,38^

Figure 3 shows the wavelength scan of the threshold for photodissociation of the UO^+^(benzene) complex, measured in the same way as that for the U^+^(benzene) complex. Again, the ions were produced in an argon expansion to promote better cooling. The intensity of the UO^+^ ion resulting from elimination of benzene was recorded as a function of the laser wavelength. There is a small gap in the spectrum at 705–710 nm, which is near the degeneracy point for the OPO where the signal and idler beams are too close spatially to separate. In this case, the threshold occurs at 697 ± 5 nm, which corresponds to 1.78 ± 0.02 eV or 41.0 ± 0.3 kcal/mol. Again, assuming that the absorption is continuous, this corresponds to the UO^+^–benzene bond energy.

**Figure 3:**
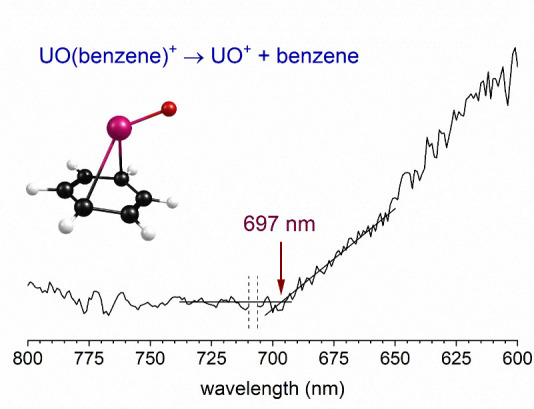
Threshold photodissociation spectrum of UO^+^(benzene) measured in the UO^+^ fragment ion channel.

Figure 4 shows the wavelength scan of the threshold for photodissociation of the UO_2_^+^(benzene) complex, measured in the same way as those for the U^+^(benzene) and UO^+^(benzene) complexes. In this case, we employed a CO_2_ expansion to improve the yield of the parent ion and to ensure cooling. The intensity of the UO_2_^+^ ion resulting from elimination of benzene was recorded as a function of the laser wavelength. In this case the data is noisier, but a threshold is evident at 720 ± 5 nm. This corresponds to a UO_2_^+^–benzene bond energy of 1.72 ± 0.02 eV or 39.7 ± 0.3 kcal/mol.

**Figure 4:**
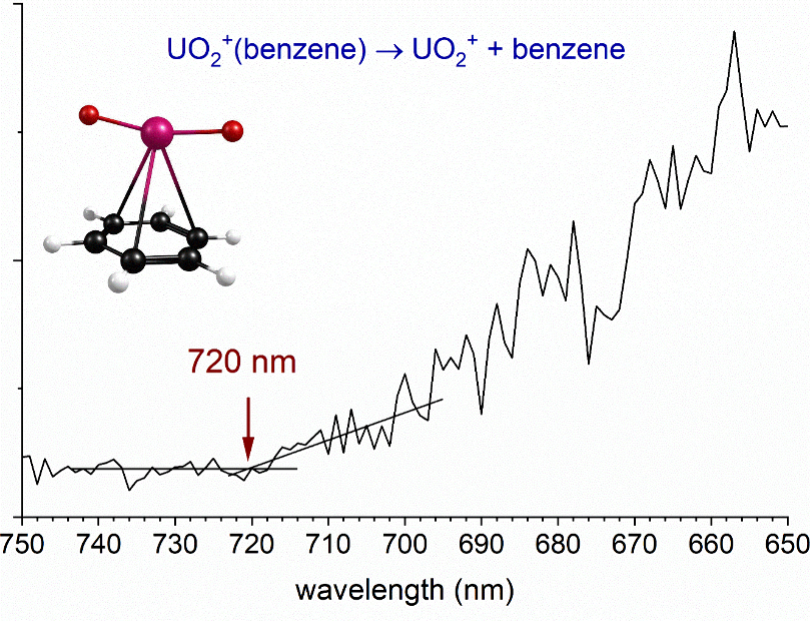
Threshold photodissociation spectrum of UO_2_^+^(benzene) measured in the UO_2_^+^ fragment ion channel.

Each of these cation-benzene complexes exhibits a threshold in their photodissociation spectra followed by continuous absorption and photodissociation at energies higher than these thresholds. These thresholds therefore provide unambiguous upper limits on the bond energies of these complexes. If the state densities are continuous in these energy regions, and the electronic states strongly coupled, these thresholds can be assigned to actual bond energies for these complexes. The high density of atomic states for U and U^+^ are well-known from atomic spectroscopy.^65,103^ It is no surprise that the density of molecular states resulting from combining U^+^ with the benzene molecule would lead to a continuous state density. UO^+^ and UO_2_^+^ have fewer valence electrons, but their high density of states have also been established with computational studies^47,58,63^ and especially with electronic and photoelectron spectroscopy.^66−72^ Again, the state density is increased by their combination with the benzene ligand. The experimentally observed continuous spectra for these various ions are therefore understandable, and because of this, these threshold energies can be assigned as the bond energies for these complexes. The resulting bond energies are presented in Table 1 where they are compared to the predictions of DFT/B3LYP/ECP60MDF/cc-pVTZ-PP theory.

**Table 1:** Energetics of Uranium-Benzene and Uranium Oxide-Benzene Cations from Computations Using Density Functional Theory and the B3LYP Functional^a^

	Mult. 2s + 1	Energy (Hartree)	Rel. Energy (kcal/mol)	BDE (kcal/mol)	TPD Exp. (kcal/mol)
benzene	1	–232.235226			
U^+^	2	–474.397528	27.98		
U^+^	4	–474.44211	0		
U^+^	6	–474.430845	7.07		
UO^+^	2	–549.770892	27.16		
UO^+^	4	–549.814178	0		
UO^+^	6	–549.672674	88.80		
UO_2_^+^	2	–625.184436	0		
UO_2_^+^	4	–625.07003	71.79		
UO_2_^+^	6	–624.906953	174.12		
U^+^(bz)	2	–706.718834	15.02	54.0	
U^+^(bz)	4	–706.742773	0	41.1	42.6 ± 0.3
U^+^(bz)	6	–706.738153	2.90	45.2	
UO^+^(bz)	2	–782.097981	7.48	57.6	
UO^+^(bz)	4	–782.109906	0	38.0	41.0 ± 0.3
UO^+^(bz)	6	–781.997398	70.60	56.2	
UO_2_^+^(bz)	2	–857.467015	0	29.7	39.7 ± 0.3
UO_2_^+^(bz)	4	–857.373254	58.84	42.7	
UO_2_^+^(bz)	6	–857.240448	142.17	61.7	

a The ECP60MDF core potential and the cc–PVTZ-PP basis set was employed for U, and the aug-cc-PVTZ basis set was employed for C, H, and O. Bond dissociation energies (BDE) are for the elimination of benzene. Experimental results are from the present threshold photodissociation (TPD) experiments.

Computational studies (see Supporting Information for full details) show that U^+^(benzene), UO^+^(benzene) and UO_2_^+^(benzene) are all cation-π complexes. Consistent with previous theory and experiments,^48−50,58,63,70,72,76,78^ the ground states of U^+^ and UO^+^ are quartets, whereas that for UO_2_^+^ is a doublet, and these same spin states carry over into the benzene complexes. U^+^(benzene) has the metal ion in a symmetric position over the benzene ring, but with a slight distortion of the planarity of the benzene. In the most stable quartet spin state, the U^+^ is located 1.95 Å from the center of the benzene ring. The angle between the plane containing C_1_, C_2_, C_3_ and C_4_ and that containing C_1_, C_5_, C_6_ and C_4_ is about 23°. This kind of deformation has been predicted for several transition metal-benzene complexes,^29^ but still awaits direct spectroscopic confirmation. UO^+^ binds to benzene through the metal and in the most stable quartet spin state the uranium atom is 2.54 Å from the center of the benzene ring. The UO^+^ oxygen is tilted at an angle (66.5°) away from the 6-fold symmetry axis. UO_2_^+^ binds through the metal with the uranium 2.80 Å from the center of the benzene ring in the most stable doublet spin state. The oxide axis is side-on to the benzene, situated on the plane that bisects opposite C–C bonds of the benzene, with a O–U–O bending angle of 164°. The computed bond dissociation energies, each considering the most stable spin state, range from 47.25 kcal/mol for the U^+^(benzene) complex, to 41.01 kcal/mol for the UO^+^ complex, to 29.71 kcal/mol for the UO_2_^+^ complex, and except for the UO_2_^+^ complex compare reasonably well with the experiments. Consistent with the experiments, the predicted bond energy is greatest for the U^+^ complex and smallest for the UO_2_^+^ complex. This trend is consistent with the greater availability of metal valence electrons to form the cation-π bonds for the less-oxidized metal ions. However, the experimental bond energies are much closer to each other than those suggested by theory. In each of these systems, the spin state of the complex is the same as that of the metal ion produced by the elimination of benzene. There is therefore no ambiguity about optical selection rules or spin changes in the fragmentation processes which could conceivably confound the thermochemistry.

A recurring theme in actinide chemistry is the role of *f* electrons in bonding compared to the role of *d* electrons in transition metal complexes. It is therefore interesting to compare these bond energies to those of some transition metal ion-benzene complexes determined previously. Transition metal cation binding energies to benzene vary widely from 30–65 kcal/mol,^35^ and the bond energies here for uranium cations fall near the middle of this range. Comparing the U^+^ (5f^3^7s^2^) configuration to transition metal ions with less than half-filled *d* shells, the bond energies for benzene complexes with Ti^+^ (3d^2^4s^1^), V^+^ (3d^4^) and Nb^+^ (4d^4^) are 61.9, 55.9, and 64.1 kcal/mol,^35^ respectively, which are all significantly higher than the U^+^(benzene) value. This comparison is oversimplified, but it is clear that the cation-π bonding interaction with uranium and its oxides involving the *5f* electrons are substantial. In the known uranium organometallics with cyclopentadiene or cyclooctatetraene, the bonding is a combination of covalent interactions with the *5f* electrons and ionic interactions. Both of these ligands accept negative charge to gain aromatic stability, leaving a more highly charged metal center, and then metal cation-ligand anion interactions enhance the bonding.^8,13^ In the present case of benzene ligands, this kind of ionic component in the bonding should not be present. U, UO and UO_2_ all have ionization energies near 6 eV,^64−72,81^ and that of benzene is 9.24 eV. Therefore, the charge should be localized on the metal center and a charge-induced dipole electrostatic interaction would augment the covalent bonding, as it does for corresponding transition metal ion-benzene complexes. However, uranium is a much larger ion than the transition metals, and so its electrostatic contribution to the bonding would be reduced compared to smaller transition metal ions that bind at shorter bond distances.

The data presented here represent the first to our knowledge for the bond energies of actinide ion organometallic complexes. Computational studies of actinide chemistry represent an active ongoing research area, and these data hopefully provide benchmark numbers to guide theory. The present computations at the DFT/B3LYP/ECP60MDF/cc-pVTZ-PP level find bond energies in reasonable agreement with theory for two out of three of these complexes. However, these complexes are small enough to encourage the application of higher-level methods for these systems. In particular, the present calculations do not include spin–orbit interaction, which may be a significant source of error. In the case of several uranium fluoride ions, for example, spin–orbit effects were found to contribute 2–10 kcal/mol to the bond energies.^56^ More advanced computational approaches including features such as spin–orbit interaction are clearly desirable. The tunable laser photodissociation threshold method employed here is applicable for a variety of actinide ion–molecule complexes, and further experimental and computational efforts in this area are planned.

## Methods

Ion molecule complexes of the form UO_*x*_^+^(benzene), (x = 0,1,2) were produced by laser vaporization^104^ of a depleted uranium rod (i.e., ^238^U) in a pulsed supersonic expansion. Expansions in argon or CO_2_ were employed to enhance ion cooling. Ions were analyzed and mass selected in a reflectron time-of-flight mass spectrometer using an instrument and methods described previously.^105,106^ Photodissociation was accomplished by excitation of selected ions in the turning region of the reflectron using a UV–visible optical parametric oscillator (OPO) laser system (Continuum Horizon II) pumped by a Nd:YAG laser (Continuum SureLite EX). The line width of this laser is about 5 cm^–1^ at visible wavelengths. The yield of specific fragment ions corresponding to the elimination of the benzene ligand was recorded as a function of the laser wavelength to record photodissociation spectra.

Computational studies on uranium and uranium oxide cation-benzene complexes were carried out with density functional theory (DFT) and the B3LYP functional as implemented in the Gaussian16 program package.^107^ These calculations used the Stuttgart/Köln fully relativistic 60 electron effective core potential and the cc-pVTZ-PP basis set for uranium,^51,53,56^ together with the aug-cc-pVTZ basis for C, H, and O.^108^

## Supporting Information Available

The full citation for ref (107) and the details of the DFT computations done, including the structures and energetics for each of the ions considered (PDF)
