# Supplementary material for: Cation-π Bonding in Actinides: UOx+(Benzene) (x = 0, 1, 2) Complexes Studied with Threshold Photodissociation Spectroscopy and Theory
Source: J Phys Chem Lett. 2025 Feb 3;16(6):1515–21. doi: 10.1021/acs.jpclett.4c03603 (PMC11831726; doi:10.1021/acs.jpclett.4c03603)
Supplement: Supplementary file 1 — jz4c03603_si_001.pdf [file jz4c03603_si_001.pdf]

Supporting Information

Cation-π Bonding in Actinides: UO_x^+(Benzene) (x = 0,1,2) Complexes Studied with Threshold Photodissociation Spectroscopy and Theory

Jason E. Colley,[1] Anna G. Batchelor,[1] B. Wade Stratton,[1] and Michael A. Duncan[1]*

[1]Department of Chemistry, University of Georgia, Athens, GA 30602

*Email: maduncan@uga.edu

Full citation for reference 107:

Frisch, M. J.; Trucks, G. W.; Schlegel, H. B.; Scuseria, G. E.; Robb, M. A.; Cheeseman, J. R.; Scalmani, G.; Barone, V.; Petersson, G. A.; Nakatsuji, H.; Li, X.; Caricato, M.; Marenich, A. V.; Bloino, J.; Janesko, B. G.; Gomperts, R.; Mennucci, B.; Hratchian, H. P.; Ortiz, J. V.; Izmaylov, A. F.; Sonnenberg, J. L.; Williams-Young, D.; Ding, F.; Lipparini, F.; Egidi, F.; Goings, J.; Peng, B.; Petrone, A.; Henderson, T.; Ranasinghe, D.; Zakrzewski, V. G.; Gao, J.; Rega, N.; Zheng, G.; Liang, W.; Hada, M.; Ehara, M.; Toyota, K.; Fukuda, R.; Hasegawa, J.; Ishida, M.; Nakajima, T.; Honda, Y.; Kitao, O.; Nakai, H.; Vreven, T.; Throssell, K.; Montgomery, J. A., Jr.; Peralta, J. E.; Ogliaro, F.; Bearpark, M. J.; Heyd, J. J.; Brothers, E. N.; Kudin, K. N.; Staroverov, V. N.; Keith, T. A.; Kobayashi, R.; Normand, J.; Raghavachari, K.; Rendell, A. P.; Burant, J. C.; Iyengar, S. S.; Tomasi, J.; Cossi, M.; Millam, J. M.; Klene, M.; Adamo, C.; Cammi, R.; Ochterski, J. W.; Martin, R. L.; Morokuma, K.; Farkas, O.; Foresman, J. B.; Fox, D. J. Gaussian 16 (Revision C.01), Gaussian, Inc., Wallingford CT, 2009.

All calculations were performed using density functional theory (DFT) with the B3LYP functional. The fully relativistic ECP60MDF Stuttgart/Cologne pseudopotential and corresponding correlation consistent triple-ζ basis set (cc-pVTZ-PP) were used for the uranium atoms. The aug-cc-pVTZ basis was used for all other atoms. The thresholds for energy and structure optimizations were set to “tight,” and all calculations used a “superfine” integration grid. The structures presented were checked for electronic wavefunction stability with the “stable=opt” keyword. All structures were free of imaginary vibrational frequencies. All electronic energies are zero-point vibrational energy (ZPVE) corrected.

Table S1. Cartesian coordinates for the optimized geometry of benzene followed by its predicted frequencies (cm^{-1}) and IR intensities (km/mol).

2s+1	E(Hartree)
1	-232.235226

Z	x	y	z
6	-1.222511000	-0.663627000	0.000000000
6	-1.185974000	0.726910000	0.000001000
6	-0.036536000	-1.390537000	-0.000001000
1	-2.108363000	1.292264000	0.000001000
1	-0.064952000	-2.472025000	-0.000001000
6	0.036537000	1.390537000	-0.000001000
6	1.185974000	-0.726910000	0.000000000
1	0.064953000	2.472026000	-0.000001000
1	2.108363000	-1.292263000	0.000003000
6	1.222510000	0.663627000	0.000000000
1	2.173315000	1.179762000	0.000000000
1	-2.173316000	-1.179761000	0.000000000

Frequency (cm^{-1})	Intensity (km/mol)	Frequency (cm^{-1})	Intensity (km/mol)
412.5984	0	1173.1774	0
412.6127	0	1197.6102	0
623.1456	0	1197.6139	0
623.1497	0	1334.2377	0
692.9827	110.1184	1387.8466	0
721.4999	0	1515.7303	6.3296
870.6926	0	1515.7322	6.33
870.6981	0	1633.6346	0
996.3698	0	1633.6432	0
996.3751	0	3157.2005	0
1013.8314	0	3167.0241	0
1022.9267	0	3167.0272	0
1030.5422	0	3182.4233	34.472
1060.4861	5.1329	3182.4249	34.4736
1060.4874	5.1331	3192.3594	0

Table S2. U^+ electronic energy for different spin states calculated using the small-core, fully relativistic ECP60MDF with the corresponding cc-pVTZ-PP basis set.

2s+1	E (Hartree)	Relative E (kcal/mol)
2	-474.397528	+28.0
4	-474.442110	+0.0
6	-474.430845	+7.1

Table S3. UO^+ electronic energy for different spin states calculated using the small-core, fully relativistic ECP60MDF with the corresponding cc-pVTZ-PP basis set for U. The aug-cc-pVTZ basis set was used for O.

2s+1	E (Hartree)	Relative E (kcal/mol)
2	-549.770892	+27.2
4	-549.814178	+0.0
6	-549.672674	+88.8

Figure S1. The optimized geometry of doublet UO^{+} followed by its predicted frequencies (cm^{-1}) and IR intensities (km/mol).

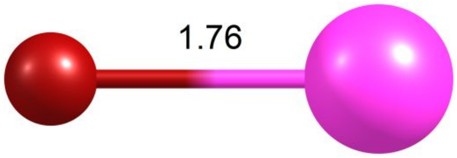

Frequency (cm^{-1})	Intensity (km/mol)
1008.2975	218.0706

Figure S2. The optimized geometry of quartet UO^+ followed by its predicted frequencies (cm^{-1}) and IR intensities (km/mol).

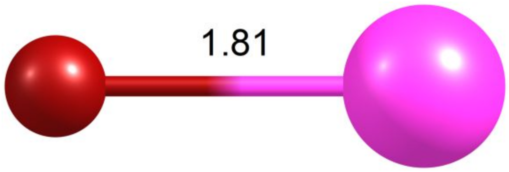

Frequency (cm^{-1})	Intensity
908.5469	214.1066

Figure S3. The optimized geometry of sextet UO^+ followed by its predicted frequencies (cm^{-1}) and IR intensities (km/mol).

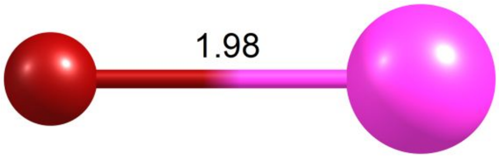

Frequency (cm^{-1})	Intensity
641.1176	102.2061

Table S3. UO_2^+ electronic energy for different spin states calculated using the small-core, fully relativistic ECP60MDF with the corresponding cc-pVTZ-PP basis set for U. The aug-cc-pVTZ basis set was used for O.

2s+1	E (Hartree)	Relative E (kcal/mol)
2	-625.184436	+0.0
4	-625.07003	+71.8
6	-624.906953	+174.1

Figure S4. The optimized geometry of doublet UO_2^+ followed by its predicted frequencies (cm^{-1}) and IR intensities (km/mol).

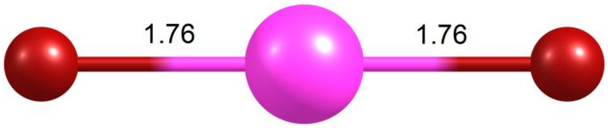

Frequency (cm^{-1})	Intensity (km/mol)
146.8918	33.971
146.9196	33.9705
931.6974	0
999.1809	384.5342

Figure S5. The optimized geometry of quartet UO_2^+ followed by its predicted frequencies (cm^{-1}) and IR intensities (km/mol).

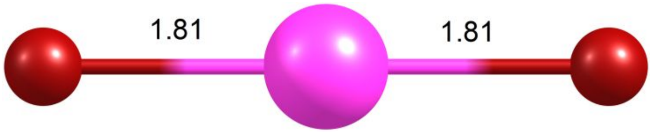

Frequency (cm^{-1})	Intensity (km/mol)
139.1871	24.7205
139.1871	24.7205
713.541	1.1391
788.5449	0

Figure S6. The optimized geometry of sextet UO_2^+ followed by its predicted frequencies (cm^{-1}) and IR intensities (km/mol).

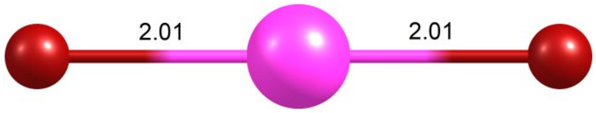

Frequency (cm^{-1})	Intensity (km/mol)
-26.2142	17.2589
57.4725	15.1808
480.0427	50.1906
575.0293	0

Table S4. $U^+(C_6H_6)$ electronic energy for different spin states calculated using the small-core, fully relativistic ECP60MDF with the corresponding cc-pVTZ-PP basis set for U. The aug-cc-pVTZ basis set was used for H and C.

2s+1	E (Hartree)	Relative E (kcal/mol)
2	-706.718834	+15.0
4	-706.742773	+0.0
6	-706.738153	+2.9

Figure S7. The optimized structure of doublet U^+(benzene).

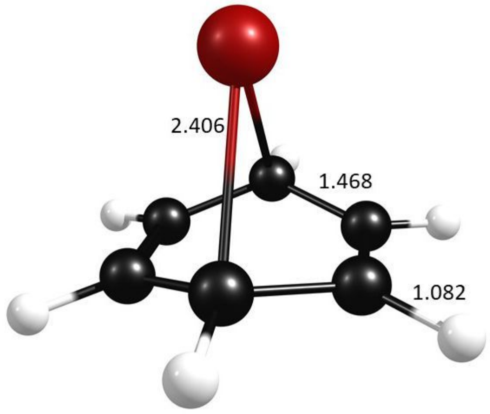

Table S5. Cartesian coordinates for the optimized geometry of doublet $U^+(C_6H_6)$ followed by its predicted frequencies (cm^{-1}) and IR intensities (km/mol).

Z	x	y	z
92	-0.675747000	-0.000004000	0.000005000
6	1.553654000	-1.208097000	-0.727209000
6	1.259426000	0.050507000	-1.427957000
6	1.556964000	-1.255028000	0.642435000
1	1.386828000	0.086482000	-2.499805000
1	1.739635000	-2.190247000	1.154438000
6	1.556913000	1.255045000	-0.642459000
6	1.259472000	-0.050495000	1.427940000
1	1.739486000	2.190292000	-1.154449000
1	1.386913000	-0.086480000	2.499783000
6	1.553654000	1.208110000	0.727188000
1	1.737699000	2.104564000	1.303813000
1	1.737696000	-2.104538000	-1.303857000

Frequency (cm^{-1})	Intensity (km/mol)	Frequency (cm^{-1})	Intensity (km/mol)
261.576	0.0344	844.1574	10.2024
264.4502	0.7652	910.2302	16.4386
269.7982	0.0248	916.3352	4.0591
340.6491	0.0002	941.526	0.0435
408.0733	14.4617	980.8761	0.8581
548.3083	6.1915	985.5457	2.9561
602.4868	0.0298	1028.524	0.0934
604.0777	10.4243	1046.4151	0.0773
756.3542	48.6451	1144.4964	0.0616
784.5	43.0644	1176.9418	0.0287
824.6777	1.5192	1287.1418	57.1933

1326.0436	0
1359.9092	0.0008
1409.2271	8.0397
1509.6477	7.3538
1574.5488	1.0428
3173.187	0.0426
3175.3049	0.0191
3182.5126	0.4658
3189.1561	0.1054
3190.6996	3.0724
3196.83	0.4284

Figure S8. The optimized structure of quartet U^+(benzene).

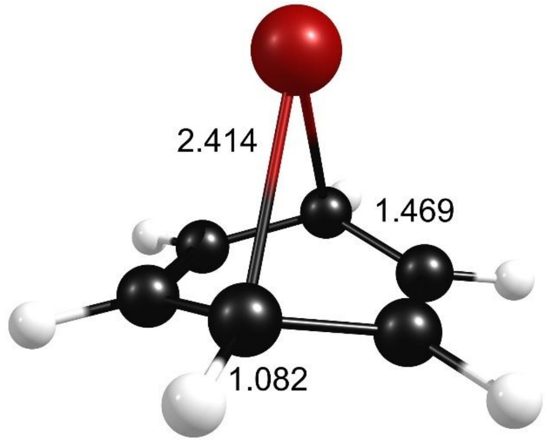

Table S6. Cartesian coordinates for the optimized geometry of quartet $U^+(C_6H_6)$ followed by its predicted frequencies (cm^{-1}) and IR intensities (km/mol).

Z	x	y	z
92	-0.678837000	-0.000003000	0.000001000
6	1.561528000	-1.231903000	0.685114000
6	1.561520000	-1.231827000	-0.685258000
6	1.266986000	-0.000080000	1.428853000
1	1.745103000	-2.148478000	-1.229450000
1	1.396292000	-0.000140000	2.501004000
6	1.266968000	0.000090000	-1.428863000
6	1.561507000	1.231836000	0.685257000
1	1.396292000	0.000156000	-2.501013000
1	1.745053000	2.148502000	1.229435000
6	1.561499000	1.231916000	-0.685118000
1	1.745062000	2.148644000	-1.229189000
1	1.745137000	-2.148619000	1.229187000

Frequency (cm^{-1})	Intensity (km/mol)	Frequency (cm^{-1})	Intensity (km/mol)
264.0417	0.9665	911.1462	17.3616
268.4052	0.0045	916.877	3.6532
268.8589	0.0033	942.9679	0
348.8226	0	983.9022	0.7898
407.1657	10.2656	987.6993	3.1033
548.1589	5.7131	1029.284	0.0879
604.0102	0	1048.1125	0
604.8561	10.4669	1145.5858	0.1439
758.8646	55.3046	1177.4632	0.0031
786.4256	44.8012	1289.1267	60.1016
826.4482	1.3469	1327.3113	0
844.2506	13.8436	1361.2279	0

1412.0569	7.1602	3182.5501	0.5436
1510.2305	7.2577	3189.1813	0.0239
1576.791	3.188	3191.9641	2.8844
3173.1265	0.0532	3197.165	0.5108
3175.1041	0		

Figure S9. The optimized structure of sextet U^+(benzene).

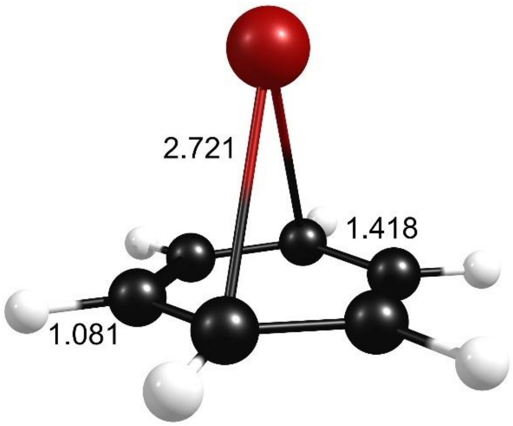

Table S7. Cartesian coordinates for the optimized geometry of sextet $U^+(C_6H_6)$ followed by its predicted frequencies (cm^{-1}) and IR intensities (km/mol).

Z	x	y	z
92	0.747870000	-0.000002000	-0.000004000
6	-1.579056000	0.000276000	1.411210000
6	-1.660647000	1.220247000	0.693987000
6	-1.660658000	-1.219975000	0.694447000
1	-1.700133000	2.157515000	1.231568000
1	-1.700133000	-2.157029000	1.232399000
6	-1.660668000	1.219977000	-0.694429000
6	-1.660687000	-1.220236000	-0.693966000
1	-1.700120000	2.157039000	-1.232373000
1	-1.700209000	-2.157498000	-1.231556000
6	-1.579118000	-0.000265000	-1.411189000
1	-1.599248000	-0.000472000	-2.490470000
1	-1.599175000	0.000469000	2.490492000

Frequency (cm^{-1})	Intensity (km/mol)	Frequency (cm^{-1})	Intensity (km/mol)
192.3669	1.2694	927.1404	4.0477
201.3888	2.2043	974.5673	2.6092
209.0985	0.5659	997.1769	0.4854
212.5297	0	1006.3025	7.0772
367.9016	0.3369	1034.007	0.2987
588.9045	0.0255	1045.3749	0.4093
610.0292	0	1114.5641	0
637.6913	1.2787	1181.5375	0.0609
760.5746	49.1527	1192.3416	2.4101
850.5476	2.0858	1346.7158	8.0829
895.4911	0.1418	1372.6232	0
924.4873	0	1432.5076	0

1477.304	10.3403	3193.7458	0.005
1498.7214	11.1024	3197.21	6.5932
1583.4324	16.7238	3208.7793	11.0121
3182.6794	0	3211.9216	4.4214
3183.0477	0.4869		

Table S8. $UO^+(C_6H_6)$ electronic energy for different spin states calculated using the small-core, fully relativistic ECP60MDF with the corresponding cc-pVTZ-PP basis set for Ur54tc x4. The aug-cc-pVTZ basis set was used for H, C, and O.

2s+1	E (Hartree)	Relative E (kcal/mol)
2	-782.097981	+7.5
4	-782.109906	+0.0
6	-781.997398	+70.6

Figure S10. The optimized structure of doublet UO^+(benzene).

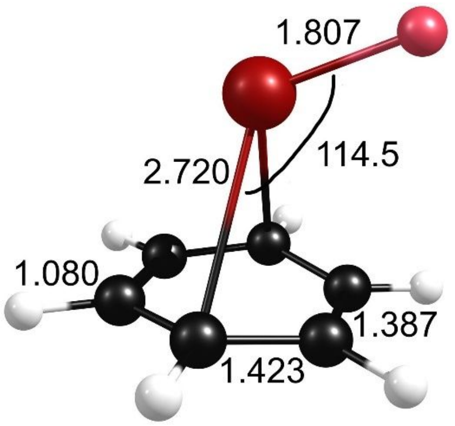

Table S9. Cartesian coordinates for the optimized geometry of doublet $UO^+(C_6H_6)$ followed by its predicted frequencies (cm^{-1}) and IR intensities (km/mol).

Z	x	y	z
92	0.636013000	-0.208889000	0.000034000
8	1.784904000	1.185556000	-0.000014000
6	-1.484577000	1.430525000	0.691759000
6	-1.484677000	1.428874000	-0.694777000
6	-1.650121000	0.213588000	1.411867000
1	-1.320535000	2.350660000	-1.234998000
1	-1.680191000	0.222086000	2.491592000
6	-1.650529000	0.210227000	-1.412000000
6	-2.043568000	-0.950556000	0.694008000
1	-1.680987000	0.216212000	-2.491732000
1	-2.323085000	-1.844561000	1.235580000
6	-2.043833000	-0.952182000	-0.691279000
1	-2.323529000	-1.847470000	-1.230637000
1	-1.320273000	2.353581000	1.229753000

Frequency (cm^{-1})	Intensity (km/mol)	Frequency (cm^{-1})	Intensity (km/mol)
38.5875	15.032	751.2238	36.0416
106.3124	11.8946	841.5183	11.4315
183.3597	1.1663	887.6314	32.2406
186.103	0.1593	893.2431	238.292
200.7675	0.2494	918.0754	13.7046
349.858	0.0198	962.7062	3.3234
359.2363	0.4213	975.8452	0.4903
587.8963	0.1903	994.897	8.0645
609.2079	0.0216	1005.3838	2.5141
644.9841	1.0756	1032.7657	0.1686

1040.1338	0.1137	1496.7386	12.9206
1113.2446	0.3877	1576.5153	28.7675
1178.5901	0.3784	3175.3804	0.0139
1192.8383	4.4801	3187.0186	0.1917
1329.9314	10.1732	3188.6639	0.0154
1371.1487	0.0007	3197.682	3.2581
1448.6632	0.1811	3202.2691	7.9102
1470.7996	10.4491	3207.5851	3.2618

Figure S11. The optimized structure of quartet UO^+(benzene).

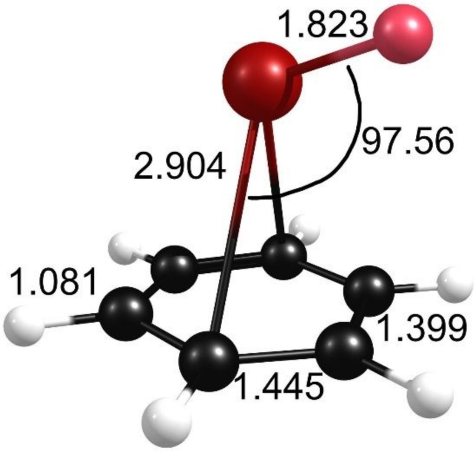

Table S10. Cartesian coordinates for the optimized geometry of quartet $UO^+(C_6H_6)$ followed by its predicted frequencies (cm^{-1}) and IR intensities (km/mol).

Z	x	y	z
92	0.676168000	-0.212849000	0.000007000
8	1.703074000	1.293341000	-0.000007000
6	-1.620332000	0.902544000	1.211652000
6	-1.508431000	1.592890000	0.000047000
6	-1.950447000	-0.473369000	1.211114000
1	-1.285605000	2.650174000	0.000087000
1	-2.103920000	-0.991835000	2.147414000
6	-1.620271000	0.902640000	-1.211611000
6	-2.158941000	-1.138207000	-0.000074000
1	-1.516147000	1.434288000	-2.147174000
1	-2.453852000	-2.179000000	-0.000114000
6	-1.950341000	-0.473285000	-1.211208000
1	-2.103727000	-0.991687000	-2.147557000
1	-1.516261000	1.434115000	2.147264000

Frequency (cm^{-1})	Intensity (km/mol)	Frequency (cm^{-1})	Intensity (km/mol)
36.61	14.4301	750.2623	44.2133
111.321	12.5086	868.5243	312.8706
139.6455	2.6006	892.0611	2.094
149.2054	0.1193	899.9251	4.0585
191.2688	0.5233	941.7432	0.0069
290.9319	0.0458	980.5546	2.597
411.8454	0.3614	1015.1949	2.0257
603.4454	0.0151	1017.5597	0.8034
611.9114	0.1523	1024.1635	4.5652
682.0488	0.7426	1037.7808	0.177

1052.8392	0.8424	1525.1899	0.0177
1166.612	0.3034	1557.2728	19.3925
1180.9244	3.4844	3179.2684	0.2097
1182.6418	0.0854	3185.5879	0.0127
1327.4011	0.9969	3189.0883	1.2291
1383.2104	0.0011	3197.6198	4.0463
1492.1457	14.8379	3199.0994	3.3428
1500.1309	20.5546	3208.7766	3.4101

Figure S12. The optimized structure of sextet UO^+(benzene).

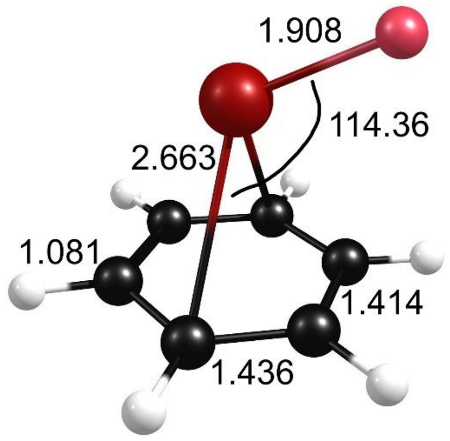

Table S11. Cartesian coordinates for the optimized geometry of sextet $UO^+(C_6H_6)$ followed by its predicted frequencies (cm^{-1}) and IR intensities (km/mol).

Z	x	y	z
92	0.579922000	-0.217979000	-0.000001000
8	1.837519000	1.216639000	0.000002000
6	-1.377903000	1.441825000	0.717905000
6	-1.377925000	1.441725000	-0.718081000
6	-1.618988000	0.248834000	1.427523000
1	-1.215082000	2.367996000	-1.248488000
1	-1.650730000	0.258085000	2.507822000
6	-1.619031000	0.248640000	-1.427522000
6	-1.848464000	-0.957074000	0.725092000
1	-1.650806000	0.257738000	-2.507821000
1	-2.088240000	-1.865766000	1.258265000
6	-1.848485000	-0.957176000	-0.724912000
1	-2.088279000	-1.865941000	-1.257952000
1	-1.215041000	2.368168000	1.248180000

Frequency (cm^{-1})	Intensity (km/mol)	Frequency (cm^{-1})	Intensity (km/mol)
13.1634	10.2786	601.0037	1.4002
107.8784	1.4774	730.7673	86.8082
225.3297	1.5653	788.8517	3.3742
234.7671	0.2901	811.4439	0.7933
236.6094	0.1745	864.1761	0.731
371.7305	67.3058	888.7795	6.3022
374.7181	0.0044	925.153	33.2596
431.6191	108.9283	931.5888	1.4462
541.5174	0.5951	960.911	3.4095
576.4902	0.2052	1011.7113	8.8082

1019.8467	1.1669	1441.4128	21.1008
1091.9978	4.2016	1469.3571	2.8869
1124.9124	39.4576	3187.9056	0.4081
1171.5828	0.8985	3189.3122	0.7758
1360.7614	0.032	3197.2089	2.4681
1370.9244	3.2469	3205.1724	1.6724
1413.1691	3.4804	3206.9201	6.2745
1424.1152	91.1151	3215.4748	8.971

Table S12. $UO_2^+(C_6H_6)$ electronic energy for different spin states calculated using the small-core, fully relativistic ECP60MDF with the corresponding cc-pVTZ-PP basis set for U. The aug-cc-pVTZ basis set was used for H, C, and O.

2s+1	E (Hartree)	Relative E (kcal/mol)
2	-857.467015	+0.0
4	-857.373254	+58.8
6	-857.240448	+142.2

Figure S13. The optimized structure of doublet UO_2^+(benzene).

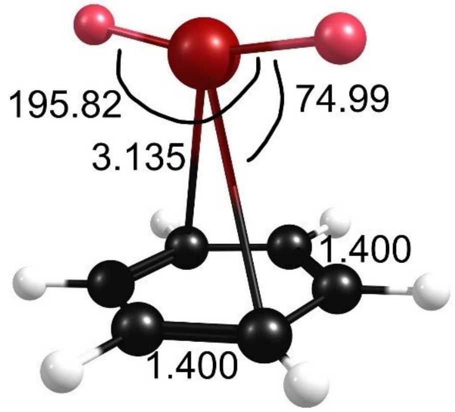

Table S13. Cartesian coordinates for the optimized geometry of doublet $UO_2^+(C_6H_6)$ followed by its predicted frequencies (cm^{-1}) and IR intensities (km/mol).

Z	x	y	z
92	-0.757079000	-0.000005000	0.000003000
8	-1.002650000	-1.768397000	0.000000000
8	-1.002753000	1.768374000	0.000002000
6	2.048233000	-1.213947000	0.696961000
6	2.048231000	-1.214464000	-0.696085000
6	2.019742000	0.000531000	1.393093000
1	2.059929000	-2.150224000	-1.236684000
1	2.030467000	0.000931000	2.475271000
6	2.019702000	-0.000501000	-1.393105000
6	2.048228000	1.214494000	0.696071000
1	2.030383000	-0.000899000	-2.475284000
1	2.059945000	2.150254000	1.236672000
6	2.048190000	1.213978000	-0.696975000
1	2.059892000	2.149340000	-1.238266000
1	2.059931000	-2.149310000	1.238249000

Frequency (cm^{-1})	Intensity (km/mol)	Frequency (cm^{-1})	Intensity (km/mol)
17.1776	0	617.7261	0.0416
61.3519	1.5048	710.3973	0.0413
98.0181	2.8185	753.0062	102.4021
130.9279	4.5311	887.6583	38.0998
137.3162	25.1262	910.5126	9.9017
145.3363	0.1467	923.2939	0.3474
195.5533	55.6524	944.4594	300.8541
407.4615	0.6481	1001.8683	2.5332
412.2604	0	1030.5936	0
616.1078	0	1030.937	0.1757

1030.9912	0.0966	1511.5551	24.8474
1047.2866	2.8944	1606.333	0
1054.223	0.0726	1619.2062	0.3432
1058.5908	1.3748	3177.222	0.0451
1185.2973	0.166	3180.2951	0.1163
1200.0867	0	3194.8564	0
1206.06	0.0862	3197.8511	1.6707
1325.3984	1.6857	3205.055	3.675
1391.8468	0	3208.373	0.021
1507.8781	19.9603		

Figure S14. The optimized structure of quartet UO_2^+(benzene).

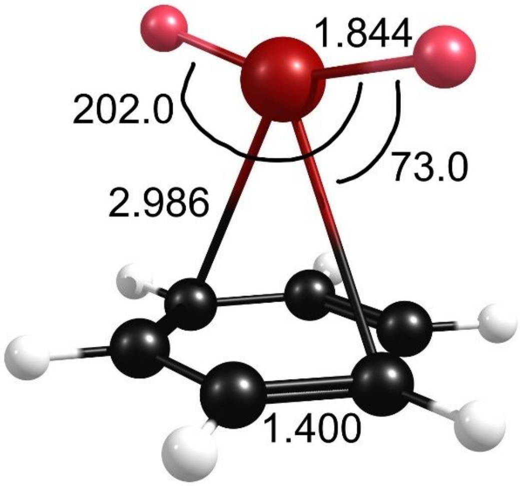

Table S14. Cartesian coordinates for the optimized geometry of quartet-$UO_2^+(C_6H_6)$ followed by its predicted frequencies (cm^{-1}) and IR intensities (km/mol).

Z	x	y	z
92	-0.696953000	-0.000005000	-0.000001000
8	-1.048873000	-1.810209000	0.000002000
8	-1.048975000	1.810176000	-0.000001000
6	1.917609000	-0.700568000	1.211902000
6	1.939608000	-1.400660000	-0.000157000
6	1.917599000	0.700330000	1.212059000
1	1.945208000	-2.480789000	-0.000277000
1	1.928563000	1.243603000	2.147119000
6	1.917620000	-0.700296000	-1.212056000
6	1.939582000	1.400695000	0.000160000
1	1.928602000	-1.243568000	-2.147116000
1	1.945164000	2.480824000	0.000281000
6	1.917603000	0.700602000	-1.211898000
1	1.928569000	1.244086000	-2.146835000
1	1.928580000	-1.244052000	2.146839000

Frequency (cm^{-1})	Intensity (km/mol)	Frequency (cm^{-1})	Intensity (km/mol)
17.8786	0	402.7126	0
130.7944	19.0787	411.845	0.0185
134.144	1.0676	610.6694	0.7473
145.2611	2.9739	612.7704	0
154.4848	2.2761	694.2283	2.7825
175.1107	2.0539	710.5766	6.1887
190.0285	30.0531	740.8133	3.7862

758.8548	106.4836	1335.907	0.0349
915.3153	0.5988	1390.5121	0
921.3374	1.6844	1503.7034	30.9914
994.4984	4.81	1507.5989	19.6597
1021.0038	0	1591.8608	0.0001
1030.4666	0.2664	1593.2218	24.3947
1033.4315	0.0655	3184.3598	0.0746
1044.1409	0.4642	3187.0919	0
1050.0449	2.7721	3194.5774	0.1792
1053.7492	0.0904	3197.2188	5.1329
1188.4723	0.001	3211.193	8.4194
1195.5906	4.3332	3213.4185	0.0022
1200.55	0		

Figure S5. The optimized structure of sextet UO_2^+(benzene).

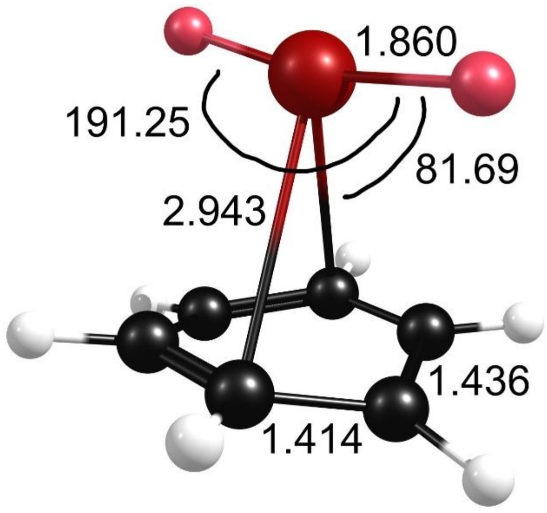

Table S15. Cartesian coordinates for the optimized geometry of sextet $UO_2^+(C_6H_6)$ followed by its predicted frequencies (cm^{-1}) and IR intensities (km/mol).

Z	x	y	z
92	-0.721386000	0.000006000	-0.000001000
8	-0.903664000	-1.850605000	0.000016000
8	-0.903578000	1.850627000	0.000006000
6	2.065764000	-1.394564000	0.000013000
6	1.850613000	-0.680669000	-1.258421000
6	1.850615000	-0.680633000	1.258436000
1	1.762119000	-1.242956000	-2.178287000
1	1.762127000	-1.242896000	2.178317000
6	1.850615000	0.680601000	-1.258440000
6	1.850634000	0.680637000	1.258418000
1	1.762120000	1.242867000	-2.178318000
1	1.762158000	1.242926000	2.178285000
6	2.065797000	1.394528000	-0.000019000
1	2.286415000	2.450074000	-0.000036000
1	2.286317000	-2.450123000	0.000030000

Frequency (cm^{-1})	Intensity (km/mol)	Frequency (cm^{-1})	Intensity (km/mol)
53.4328	0	228.1687	1.1385
90.3796	4.4605	300.8001	0
106.2883	1.8661	313.1401	0.0976
122.8905	0.047	533.2536	18.1899
128.4549	19.7922	554.5663	0
157.1847	20.5101	589.6088	1.6972
170.0908	1.3804	610.6651	47.6959

676.2771	5.9391
712.4476	36.1801
761.3956	5.9768
808.9884	63.1925
842.4175	1.1034
880.347	0
919.2462	14.1093
980.0293	4.3043
988.3441	0
1015.2619	0.268
1028.295	0.3686
1170.5415	0.2364
1194.1062	11.6618
1322.5897	0
1357.7218	0
1358.6219	5.9105
1413.2007	24.7381
1551.3951	2.3498
1608.7037	81.4456
3181.8351	0
3183.6227	4.8378
3196.0564	1.105
3196.9067	6.7035
3225.0395	16.6966
3227.4904	12.0351
